# Molecular Advances in Microbial Metabolism 2.0

**DOI:** 10.3390/ijms25021361

**Published:** 2024-01-22

**Authors:** Rosa María Martínez-Espinosa

**Affiliations:** 1 Department of Biochemistry, Molecular Biology, Edaphology and Agricultural Chemistry, Faculty of Science, University of Alicante, Carretera San Vicente del Raspeig s/n, 03690 San Vicente del Raspeig, Alicante, Spain; rosa.martinez@ua.es; 2 Multidisciplinary Institute for Environmental Studies “Ramón Margalef”, University of Alicante, Ap. 99, E-03080 Alicante, Alicante, Spain

The advances in molecular biology techniques and omics approaches have made it possible to take giant steps in applied research in life sciences. In this context, microbiology and issues related to microbial metabolism have constituted one of the most popular fields, especially research focused on the application of microorganisms in biotechnology [1,2]. This justifies the proliferation of research studies and reviews on this topic, and this Special Issue contributes to this topic as a continuation of our previous Special Issue, “Molecular Advances in Microbial Metabolism”.

Microbial metabolism is one of the main driving forces behind the development and maintenance of the biosphere and most biotechnological-based processes [3]. Due to the relevance of these metabolic pathways, this Special Issue focuses on molecular mechanisms underlying microbial metabolism, not only to improve the knowledge around processes carried out by microbes to obtain the energy and nutrients required to live and reproduce, but also to shed light on microbial evolution, the lives of microbes in extreme environments and the potential applications of metabolic pathways utilized by microbes in biotechnology and industrial processes. This multidisciplinary topic comprises several disciplines, such as microbiology, molecular biology, genetics, chemistry, microbial ecology, biochemistry, biophysics, and all omics-based sciences which offer insights into the impact that modern technologies have on microbiological research today.

Overall, five papers have been included in this Special Issue, all of which are focused on bacteria as model organisms. To summarise the main findings of the works published in this Special Issue, data from traditional molecular biology studies will be followed by the data reported in these papers using new-generation approaches (mostly based on omics). Finally, new advances reported from potential applications of some bacterial species are summarized.

Related to more classical studies focusing on plasmids and considering that plasmids have a key role in controlling bacterial phenotype and chromosomal gene expression, Kosiorek and coworkers explore the role of single plasmids in *Lactococcus lactis* IL594 [4] thanks to global comparative phenotypic analyses combined with transcriptomic studies in plasmid-free *L. lactis* IL1403, multiplasmid *L. lactis* IL594, and its single-plasmid derivatives. Comparative transcriptomics showed significant variation in the expression levels of up to 189 chromosomal genes due to the presence of single plasmids and 435 unique chromosomal genes that were resultant of the activity of all plasmids, which may suggest that the observed phenotypic changes are not only due to the direct action of their genes but also originate from indirect actions through crosstalk between plasmids and the chromosome. The data obtained suggest that plasmid maintenance leads to the development of important mechanisms of global gene regulation that provide changes in the central metabolic pathways and adaptive properties of *L. lactis* and probably of other bacteria [4].

Regarding omics-based sciences and large database analysis, Blázquez and coworkers [5] provide new insights into the metabolic features of *Bacillus subtilis* by using multistrain genome-scale metabolic modelling. *B. subtilis* is a very well-described bacteria with several potential uses in industry that have been identified and upscaled. For this reason, the improvement in the characterization of its metabolic capabilities as well as the identification of new sources of nutrients for the growth of this bacteria at a large scale reveal developing processes based on the use of *B. subtilis* as an objective for laboratories and companies. Genome-scale metabolic models (GEMs) are powerful tools for predicting the metabolic capabilities of a given organism and constitute the main approach used by Blazquez and colleagues to construct a manually curated genome-scale model for *B. subtilis* (iBB1018). The model was validated by monitoring the growth performance of the cells and carbon flux distribution. Thanks to the model accurate predictions of carbon source utilization, up to 28 metabolites are identified as potential novel carbon sources [5]. The constructed model is further used as a tool for the construction of the panphenome of *B. subtilis* as a species. The results highlight the large metabolic versatility of the species and the relevance of the accessory metabolism as a driver of the panphenome at the species level.

GEMs were first developed from the Gram-negative bacterium *Haemophilus influenzae*, mainly due to the relevant clinical implications. However, although powerful, GEMs are unable to capture metabolomic features, making the development of a comprehensive picture of the metabolic capabilities of living beings possible. For this reason. Fernández-García and coworkers [6] explore the endometabolome of *H. influenzae* Rd KW20 by performing a multiplatform MS-based metabolomics approach combining LC-MS, GC-MS and CE-MS. Thanks to this strategy, direct evidence of 15–20% of the endometabolome present in current *H. influenzae* GEMs is obtained, showing that polar metabolite pools are interconnected through correlating metabolite islands. In addition, 18 metabolites not previously included in *H. influenzae* GEMs, including the antimicrobial metabolite cyclo (Leu-Pro), are accurately identified. Additionally, it is possible to characterize the quantitative composition of the phospholipidome of *H. influenzae*, which makes it possible to estimate the abundance of low-level species, permitting the expansion of the phospholipidome characterization through predictive probabilistic modelling [6].

In terms of the potential applications in biotechnology of microorganisms showing genuine metabolic capabilities, works conducted by Wang and coworkers [7] and Biello and coworkers [8] are good examples of how so-called “basic science” provides insights to develop “applied sciences”. The work conducted by Wand and colleagues describes the role and the synergic effect of the genes *paaZ* and *ech* in the biosynthesis of the iron scavenger 7-hydroxytropolone (7-HT) in *Pseudomonas donghuensis* HYS. 7-HT belongs to a class of natural products showing several biological activities of interest in pharmacy and medicine [7]. On the other hand, Biello and coworkers conducted a proteomic analysis complemented by qRT-PCR analysis and the determination of analytes of arsenic resistance during cyanide assimilation by *Pseudomonas pseudoalcaligenes* CECT 5344, whose results confirm that this strain could be used for the design and development of bioremediation strategies for industrial wastes co-contaminated with cyanide and arsenic [8]. In summary, these two works based on biochemistry, molecular biology and omics offer solutions and/or alternatives to improve biotechnological processes related to the synthesis of molecules of interest (as it is 7-HT) or the treatment of sites polluted by contaminants like cyanide, which is one of the main concerns in regions where jewellery constitutes a relevant industrial activity.

With these works, the authors contributing to this Special Issue provide advances in the understanding of microbial metabolism and their potential applications in biotechnology. They also provide insights into large-scale models to analyze genome sequences. The use and implementation of omics approaches provide a vast amount of heterogeneous data that require the adaptation or development of computational tools for a system-wide interrogative analysis of microorganisms or consortia to be studied in detail in the future [2]. In this context, the utilization of multi-omics tools concomitantly with genome-scale metabolic and mathematical models and better bioinformatics tools constitutes one of the main challenges for the next few years [9,10]. On the other hand, a better understanding of how metabolomics allows the characterization of the network topologies of metabolism and its regulation networks will elucidate the control of metabolic function and will contribute to understanding the molecular basis of higher-order microbial metabolism phenomena [11]. Finally, considering the promising and genuine metabolism of extremophilic microorganisms and the lack of knowledge about their metabolism compared to mesophilic microorganisms, more research should be conducted in this area [12,13]. In this sense, something as simple as cultivating 100% of the strains of extremophiles identified continues to be a challenge for microbiology, which must be addressed in the coming years [14].
